# Supplementary material for: A Global Screen for Assembly State Changes of the Mitotic Proteome by SEC-SWATH-MS
Source: Cell Syst. 2020 Feb 26;10(2):133–155.e6. doi: 10.1016/j.cels.2020.01.001 (PMC7042714; doi:10.1016/j.cels.2020.01.001)

Q9HB40 | RISC_HUMAN | SCPEP1 RISC SCP1 MSTP034 UNQ265/PRO302
Monomer MW [kDa]: 50.831 Monomer expected elution fraction: 46

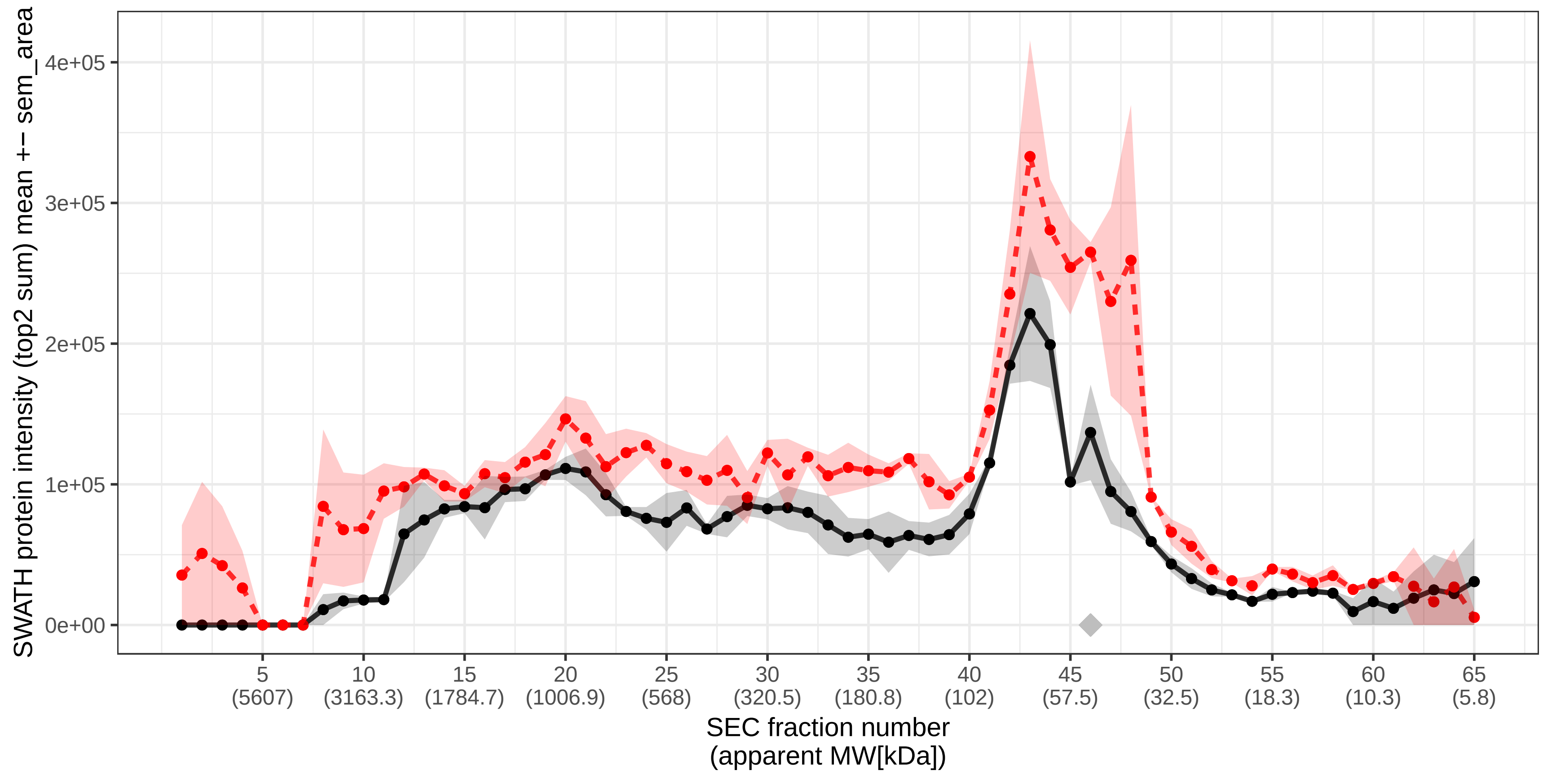

Supplement: Data S1. SEC-SWATH-MS Protein Chromatograms, Related to Figure 1 [file mmc6.zip › SECchrom_Q9HB40_RISC_HUMAN_SCPEP1_RISC_SCP1_MSTP034_UNQ265_.pdf]
